# Structure of *Clostridium leptum* carboxyspermidine decarboxylase and comparison to homologs prevalent within the human gut microbiome

**DOI:** 10.1107/S2053230X25000482

**Published:** 2025-01-31

**Authors:** Savannah J. Jones, Dawson J. Bell, Jeffrey S. McFarlane

**Affiliations:** a Department of Chemistry and Biochemistry Fort Lewis College Durango CO81301 USA; Hampton University, USA

**Keywords:** polyamines, spermidine, gut microbiome, carboxyspermidine decarboxylase

## Abstract

Polyamines are key signalling and substrate molecules that are made by all organisms. The polyamine known as spermidine is typically made by spermidine synthase, but in many bacterial species, including 70% of human gut microbes, carboxyspermidine decarboxylase (CASDC) performs the terminal step in the production of spermidine. An X-ray crystal structure of CASDC from the human gut microbe *Clostridium leptum* has been solved by molecular replacement at a resolution of 1.41 Å. CASDC is a homodimer, with each monomer composed of two domains: a β/α-barrel pyridoxal 5′-phosphate-binding domain that forms most of the active site and a β-barrel domain that extends the dimeric interface and contributes to the active site of the opposing monomer. We performed a structural comparison of CASDC enzymes for 15 common genera within the human gut flora. This analysis reveals structural differences occurring in the β6/β7 loop that acts as a ‘flap’ covering the active site and in the α9/β12 loop that is connected to the α9 helix which is thought to select substrates by their chain length. This structural analysis extends our understanding of a key enzyme in spermidine biosynthesis in many bacterial species.

## Abstract 2

A high-resolution structure of carboxyspermidine decarboxylase, a member of the PLP-dependent β/α-barrel-fold decarboxylase family, is analyzed.

## 1. Introduction

**Figure 1:**
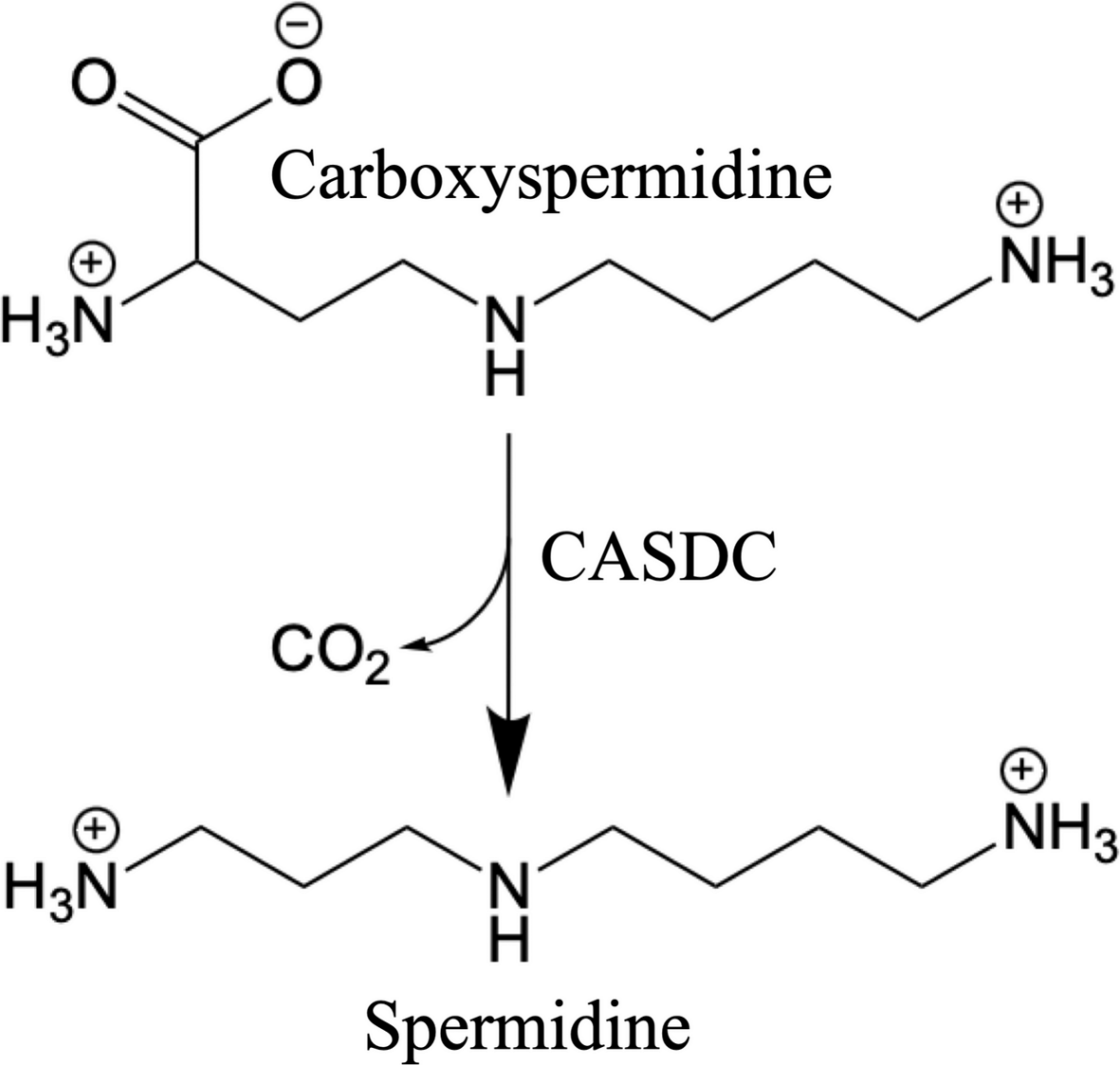
Carboxyspermidine decarboxylase (CASDC) reaction scheme.

The production of polyamines is an essential and ubiquitous cellular function due to the role that polyamines play in key processes such as translation elongation, gene expression and biofilm formation (Shah & Swiatlo, 2008 ▸; Michael, 2016 ▸; Ramos-Molina *et al.*, 2019 ▸). Most prokaryotic and eukaryotic organisms share a semi-conserved biosynthetic pathway for the production of the polyamine spermidine which uses decarboxylated *S*-adenosyl-l-methionine as a propylamine donor (Pegg & Michael, 2010 ▸). However, approximately 70% of bacterial species within the human gut microbiome make the polyamine spermidine (or, less frequently, norspermidine) through a distinct two-enzyme biosynthetic pathway (Sugiyama *et al.*, 2017 ▸). Carboxyspermidine dehydrogenase is the first enzyme in the pathway and uses NADPH to perform a reductive condensation of aspartate semialdehyde and putrescine (1,4-diaminobutane) to produce carboxyspermi­dine (Lee *et al.*, 2023 ▸). Carboxyspermidine decarboxylase (CASDC) is a pyridoxal 5′-phosphate (PLP)-dependent enzyme that removes the carboxylate of carboxyspermidine to produce spermidine (Deng *et al.*, 2010 ▸; Fig. 1 ▸).

A 1.90 Å resolution structure of carboxynorspermidine decarboxylase, which produces norspermidine, has previously been characterized from the bacterium *Campylobacter jejuni* (PDB entry 3n29; Deng *et al.*, 2010 ▸). This family of enzymes forms an external aldimine between PLP and the carboxy­spermidine substrate, with PLP acting as an electron sink during decarboxylation. CASDC enzymes are members of the β/α-barrel-fold type IV amino-acid decarboxylase family (Mehta & Christen, 2000 ▸). These enzymes are composed of two domains. The core catalytic domain is composed of a β/α-barrel. A secondary domain is primarily β-barrel and serves to extend the dimerization interface and enclose one surface of the active site. Two common homologs within the family are ornithine decarboxylase, a polyamine-biosynthetic enzyme that produces putrescine from ornithine in eukaryotes (Almrud *et al.*, 2000 ▸), and diaminopimelate decarboxylase, which forms l-lysine through the decarboxylation of diaminopimelate in many bacterial species (Ray *et al.*, 2002 ▸).

Here, we report a 1.41 Å resolution X-ray structure of CASDC from *Clostridium leptum* (ClCASDC), a member of the human gut flora. This structure resolves an active-site flap that was absent from the *C. jejuni* CASDC structure and demonstrates a conformational flip for the active-site residue Cys306, which may have implications for understanding the mechanism of CASDC. A comparison of ClCASDC, *C. jejuni* CASDC and homologs from other gut constituents was completed to reveal active-site and domain variation within the CASDC family.

## 2. Materials and methods

### 2.1. Macromolecule production

The gene sequence for carboxyspermidine decarboxylase from *C. leptum* was obtained from the NCBI database under accession No. EDO61992.1. The sequence was codon-optimized for expression in *Escherichia coli* by GenScript and ligated into a pET-28b vector encoding an N-terminal hexahistidine-affinity tag. The resulting plasmid was transformed into New England Biolabs *E. coli* BL21 (DE3) cells for expression (Table 1 ▸).

To purify ClCASDC, the cells were expressed in a baffled flask with 1 l LB Miller broth containing 50 µg ml^−1^ kanamycin inoculated with 10 ml of overnight culture and grown to an *A*_600_ of 0.6–0.8 at 37°C (∼3 h). Cultures were induced with 200 µl of 1 *M* isopropyl β-d-1-thiogalactopyranoside (IPTG) and were grown for 19 h at 25°C. The cells were centrifuged at 3025*g* for 10 min. The pellet was resuspended in buffer *A* (25 m*M* Tris pH 8, 300 m*M* NaCl, 50 m*M* imidazole). The cells were lysed by sonication using a Branson 150 sonicator with a microtip at 50% amplitude for 10 min using a 15 s pulse, 45 s pause cycle. The cell lysates were centrifuged at 23 700*g* for 1 h and the lysate was injected onto a nickel-chelating Sepharose column (Cytiva 6 Fast-Flow resin) equilibrated with five column volumes of buffer *A*. The protein eluted from the nickel-chelating Sepharose column in 100% buffer *B* (25 m*M* Tris pH 8, 300 m*M* NaCl, 300 m*M* imidazole) and flowed directly onto a Cytiva HiPrep 26/10 desalting column pre-equilibrated with buffer *C* (25 m*M* HEPES pH 6.8, 150 m*M* NaCl, 10% glycerol). ClCASDC was concentrated using an Amicon Ultra 15 concentrator with a 10 000 kDa molecular-weight cutoff to a final concentration of 4.5 mg ml^−1^ as measured by *A*_280_ assay (ɛ = 40 340 *M*^−1^ cm^−1^ for ClCASDC). This preparation yields 7 mg of ClCASDC per litre of culture, which was greater than 95% pure by SDS–PAGE analysis (Supplementary Fig. S1). The purified protein was visibly yellow, with an absorbance peak at 420 nm, which was interpreted to be due to the pyridoxal 5′-phosphate (PLP) internal aldimine.

Initial crystallization screens were performed using 25 m*M* Tris pH 8 as the protein storage buffer but yielded no crystals. Thermal shift assays were performed using the Hampton Research Slice pH screen. Each 20 µl screen condition consisted of 5 µl 10 µ*M* ClCASDC containing 5× SYPRO Orange fluorescent dye, 2 µl Slice pH screen condition and 13 µl water. Thermal melt curves were measured using a Bio-Rad CFX RT-PCR and *T*_m_ values were calculated in *Excel*. Several lower pH conditions showed thermal stability similar to Tris pH 8. HEPES pH 6.8 was selected as the new storage buffer for crystallization trials, leading to several viable crystallization conditions.

### 2.2. Crystallization

Crystals were grown in sitting drops composed of a 1 µl:1 µl ratio of protein and well solution at 25°C. ClCASDC crystals were grown using freshly purified protein (unfrozen) at 4.5 mg ml^−1^ with the hexahistidine-affinity tag intact that had been pre-incubated with 100 µ*M* PLP and 5 m*M* dithiothreitol (DTT). The well solution consisted of 0.04 *M* monopotassium phosphate, 16%(*w*/*v*) PEG 8000, 20%(*w*/*v*) glycerol (Table 2 ▸). The crystals were soaked in cryoprotectant composed of well solution supplemented with 10% glycerol. The crystals were looped and flash-cooled in liquid nitrogen prior to data collection.

### 2.3. Data collection and processing

Diffraction data were collected remotely using *Blu-Ice* (McPhillips *et al.*, 2002 ▸) on beamline 12-2 at the Stanford Synchrotron Radiation Lightsource (SSRL). A full 360° of data were collected at a wavelength of 0.9795 Å with 0.15° oscillation. An image was taken every 0.2 s at a temperature of 100 K. The detector distance was 213 mm. Data were processed to 1.41 Å resolution in *XDS* (Kabsch, 2010 ▸). Statistics for data collection and processing are listed in Table 3 ▸.

### 2.4. Structure solution and refinement

The structure of ClCASDC was solved by molecular replacement using *phenix.phaser* (McCoy *et al.*, 2007 ▸) with PDB entry 3n29 chain *A* as a model without modification except for the removal of waters and ligands. The resulting log-likelihood gain (LLG) was 1527.41 and the translation-function *Z*-score (TFZ) was 16.3. The initial model was completed in *phenix.autobuild*, which placed 738 of 752 residues with *R*_free_ = 26.87% and *R*_work_ = 23.98%. Electron density corresponding to PLP was clearly visible in chains *A* and *B* following molecular replacement. Rounds of model building and refinement were completed in *Coot* (Emsley *et al.*, 2010 ▸) and *phenix.refine* (Adams *et al.*, 2010 ▸). Waters were placed by *phenix.refine* and corrected manually. Once model building was complete, PLP was added in *Coot* by replacing Lys43 with the modified lysine (code LLP). The finished model was refined to *R*_free_ = 18.9% and *R*_work_ = 15.0%. Ramachandran analysis was performed by *MolProbity* (Chen *et al.*, 2010 ▸), showing 97.95% favoured conformations with no outliers. Statistics for refinement are listed in Table 4 ▸. The ClCASDC interface was analysed using *PDBePISA* (Krissinel & Henrick, 2004 ▸). Structural alignments were performed and r.m.s.d. values were calculated in *PyMOL* version 3.0 (Schrödinger) using the *cealign* algorithm. The structure of ClCASDC was deposited in the PDB as entry 9eaf.

## 3. Results and discussion

### 3.1. Purification and crystallization

ClCASDC was expressed and purified from *E. coli* as described in Section 2. The purified protein was visibly yellow in colour and showed an absorbance peak at 420 nm corresponding to the internal aldimine form of PLP. ClCASDC was unable to be crystallized using a Tris buffer system at pH 8. Thermal shift assays were used to identify additional, stable buffer conditions. From these data, a crystallization buffer consisting of 25 m*M* HEPES pH 6.8, 150 m*M* NaCl, 10% glycerol was selected. The protein was concentrated to 4.5 mg ml^−1^ and was pre-incubated with 100 µ*M* PLP, 5 m*M* DTT. Crystals grew in a flower morphology using a well solution consisting of 0.04 *M* monopotassium phosphate, 16%(*w*/*v*) PEG 8000, 20%(*w*/*v*) glycerol.

### 3.2. Structure determination of ClCASDC 

The crystal structure of ClCASDC was determined by X-ray crystallography at a resolution of 1.41 Å. *Phenix.phaser* completed molecular replacement using chain *A* of carboxy­norspermidine decarboxylase (PDB entry 3n29) as a model. *Phenix.autobuild* placed 98% of ClCASDC residues with a final *R*_free_ value of 25.2%. Electron density corresponding to PLP was clearly visible upon initial inspection of the 2*mF*_o_ − *DF*_c_ maps following molecular replacement. Data-collection and refinement statistics can be found in Table 2 ▸. These data have been deposited with the PDB as entry 9eaf.

### 3.3. Structure and assembly of ClCASDC

**Figure 2:**
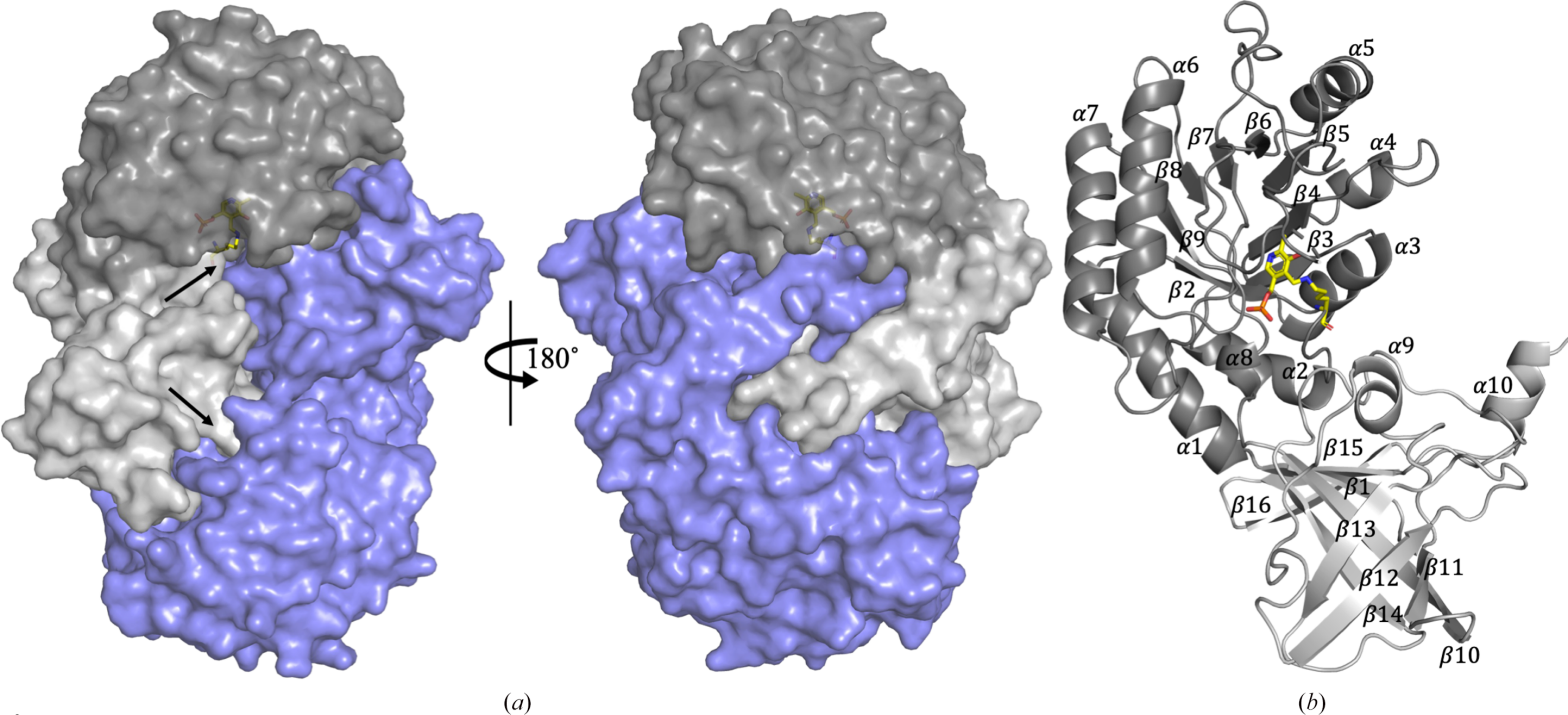
Carboxyspermidine decarboxylase structural overview. (*a*) ClCASDC dimer. Active-site channels within each monomer are indicated by black arrows. Chain *A* is shown in grey. The β/α-barrel, PLP-binding domain is shown in dark grey. The β-barrel interface domain is shown in light grey. Chain *B* is shown in blue. (*b*) Secondary-structure labelling. The chain *A* monomer is shown. Pyridoxal 5′-phosphate and Lys43 are shown with yellow carbons.

The asymmetric unit of ClCASDC contains one homodimer stabilized by an extensive interface with a surface area of 3558 Å^2^ as calculated by *PDBePISA* (Krissinel & Henrick, 2007 ▸; Fig. 2 ▸*a*). This assembly and interface surface are highly conserved in CASDC homologs (comparisons are made below).

The ClCASDC monomer contains two domains (Fig. 2 ▸*b*). The PLP-binding domain is composed of a β/α-barrel with eight β-strands and eight α-helices spanning residues 16–245. This domain contains the PLP-binding site and most of the amino-acid residues composing the active site. The second domain has eight β-strands forming a five-stranded β-barrel that extends into a β-sheet made of the three additional strands. Residues 1–15 form the β1 strand making a connection between the two domains. Residues 246–376 form the remainder of the second β-barrel domain. This domain seems to serve two functions: it extends the dimerization interface and contributes loops β2/13 and β14/15 that form a portion of the active-site pocket. As such, the two domains are tightly interconnected, with each monomer contributing amino-acid residues to the opposing active site.

ClCASDC was purified using a N-terminal hexahistidine affinity tag, and electron density for eight residues of this tag was resolved during refinement (Supplementary Fig. S2). The tags appear to originate from the N-terminus of the same monomer, but the linker density is weak and could not be modelled, so the histidine tags were designated chains *C* and *D* in the PDB file. Only the first two (chain *C*) or three (chain *D*) histidines are resolved. The remaining histidines curl out of the active site. Electron density for these tags is weaker than for the remainder of the structure, suggesting partial occupancy of the tag in the active site.

### 3.4. The active site of ClCASDC

**Figure 3:**
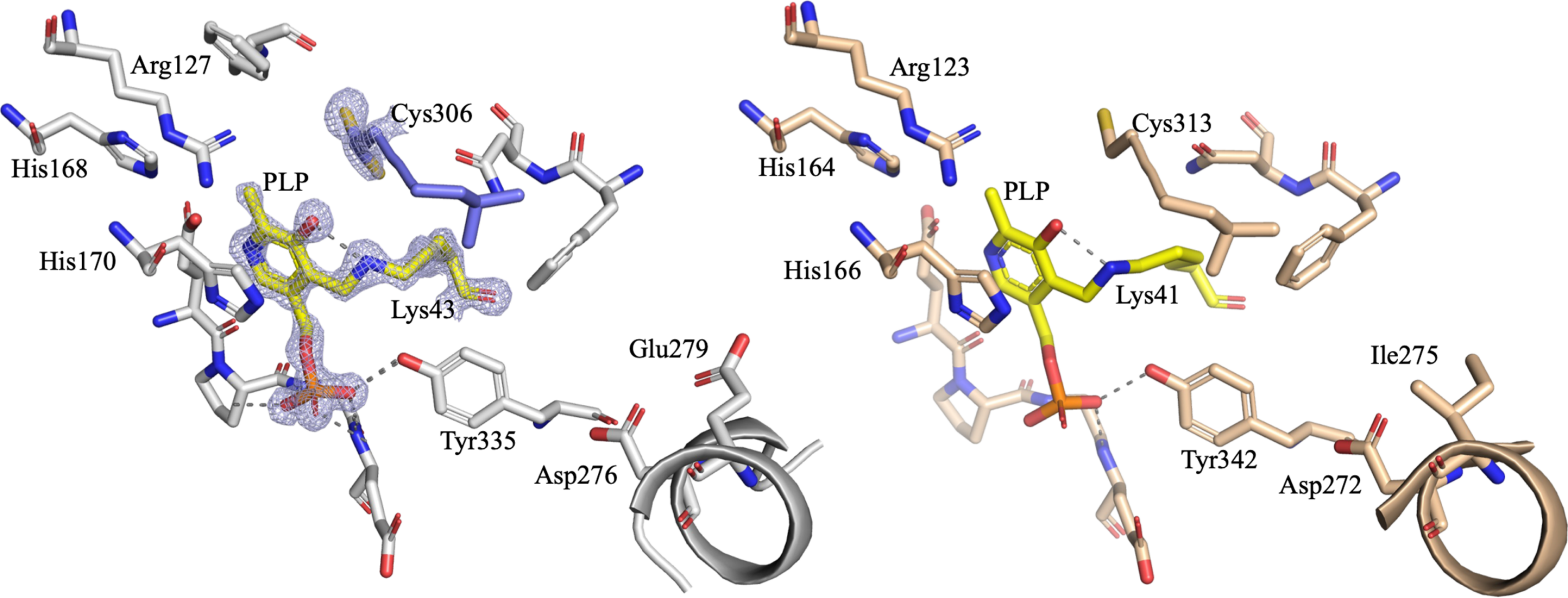
CASDC active site. Pyridoxal 5′-phosphate is shown with yellow carbons. (*a*) ClCASDC. Chain *A* is shown with grey carbons. Chain *B* is shown with blue carbons. Asp276 originates from α9, the specificity helix. Cys306 has 70% occupancy in the ‘up’ position relative to the figure orientation. Electron density is displayed as a 2*mF*_o_ − *DF*_c_ map contoured at 1.5σ. (*b*) CjCASDC (PDB entry 3n29).

Electron density corresponding to PLP was observed in the active site of both monomers of ClCASDC. PLP forms a Schiff base with Lys43 in both chains and the arrangement of amino acids within the active site is very similar to that in CjCASDC (PDB entry 3n29; Fig. 3 ▸, Supplementary Fig. S3). The 5′-phosphate is coordinated by the backbone amines of Gly236, Gly239 and Glu240, by the hydroxyl group of Tyr335 and through a water molecule stabilized by Cys173 and Glu240. His170 forms a π-stacking interaction with the pyridoxamine ring and Glu237 forms a hydrogen bond to the pyridoxamine nitrogen. Weak electron density corresponding to a glycerol was observed near the position where carboxyspermidine would be expected to bind. The opposing chain contributes residues 306–308 of the β12/13 loop and residues 343–344 of the β14/15 loop to the active site. Electron density for two conformations of Cys306 was visible. The sulfhydryl group faces away from PLP with 70% occupancy (both chains), placing the sulfur in a position in which it is unable to hydrogen-bond to any surrounding residues. The explanation for this seemingly unfavourable position may lie with the Cys306 backbone carbonyl, which appears to shift ∼1.4 Å between the two conformations. This shift alters the ability of the carbonyl oxygen to donate electrons to the pyridoxamine oxygen and Schiff-base nitrogen, which may be important to facilitate the expected proton transfer during the catalytic cycle. Helix α9 positions Asp276 to coordinate the terminal amine of carboxyspermidine. This helix, also termed the specificity helix, has previously been observed to shift ∼1.5 Å between different homologs, and its position seems to correlate with substrate length (Deng *et al.*, 2010 ▸).

### 3.5. CASDC enzymes of the human gut microbiome

The CASDH/CASDC metabolic pathway is dominant among human gut microbes, with ∼70% of gut species encoding these enzymes (Sugiyama *et al.*, 2017 ▸; S. J. Jones & J. S. McFarlane, unpublished work). As *Clostridium leptum* is a common human gut constituent, we performed a structural comparison of CASDC enzymes from the most common genera as determined by recent genomic analyses (Liu *et al.*, 2021 ▸). A UniProt search for enzymes annotated as carboxynor­spermidine decarboxylase returned 8285 entries. From this group, one enzyme was chosen from the following species as a representative either of the most common genera or of potentially pathogenic species: *Roseburia intestinalis*, *Ruminococcus flavefaciens* (RfCASDC), *Lacnospira eligens*, *Streptococcus pneumoniae*, *Blautia hansenii*, *Faecalibacterium prausnitzii*, *Clostridium leptum* (ClCASDC), *Bacteroides fragilis*, *Akkermansia muciniphila* (AmCASDC), *Parabacteroides merdae*, *Helicobacter pylori* (HpCASDC), *Prevotella intermedia*, *Allistipes finegoldii*, *Vibrio cholerae* and *Campylobacter jejuni* (CjCASDC).

**Figure 4:**
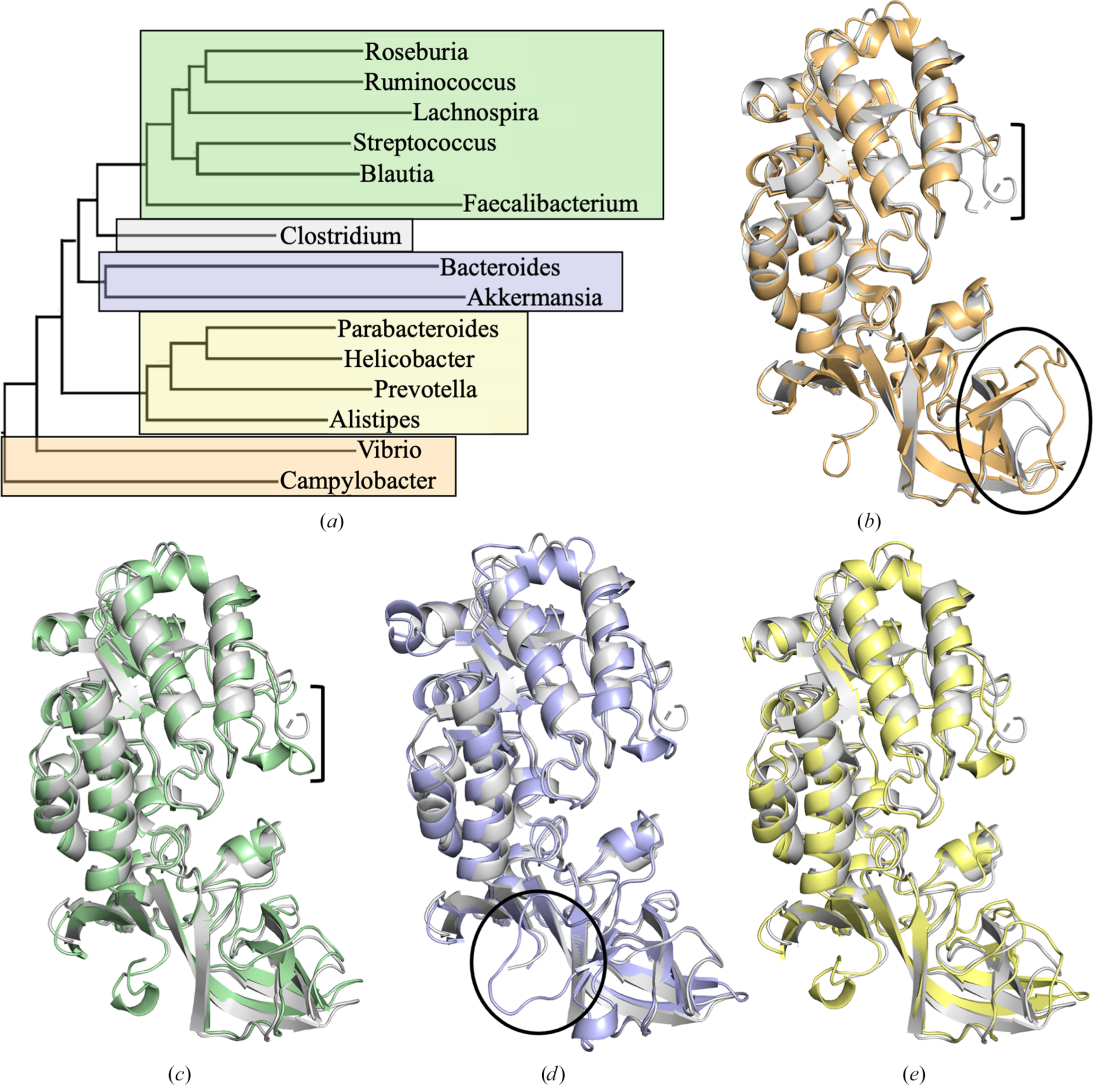
CASDC family comparison. (*a*) Dendrogram generated in *DALI* illustrating the structural relationships of CASDC enzymes from high-frequency human gut genera. Coloured blocks are for illustration and coordinate with the structural overlays. ClCASDC is displayed as a grey cartoon. (*b*) Alignment of *Campylobacter jejuni* CASDC. (*c*) Alignment of *Ruminococcus flavefaciens* CASDC. (*d*) Alignment of *Akkermansia muciniphila* CASDC. (*e*) Alignment of *Helicobacter pylori* CASD. The β6/β7 loop is indicated by brackets. The α9/β12 loop is indicated by circles.

The structure of ClCASDC, the structure of CjCASDC (PDB entry 3n29) and *AlphaFold* models of CASDC from the remaining species were compared using an all-against-all structural comparison in *DALI* (Holm, 2022 ▸). A dendrogram comparing these 15 genera was created in *DALI* (Fig. 4 ▸*a*). Using the clusters within the dendrogram, five CASDC homologs were selected for further analysis (RfCASDC, ClCASDC, AmCASDC, HpCASDC and CjCASDC). The overall similarity between ClCASDC and these homologs was high, with r.m.s.d. values ranging from 1.07 Å over 360 residues for AmCASDC to 1.89 Å over 352 residues for CjCASDC. The position of PLP within these five structures is virtually identical. However, two notable areas of difference were observed and annotated in a structural sequence alignment generated in *T-Coffee* (Di Tommaso *et al.*, 2011 ▸; Supplementary Fig. S4). The β6/7 loop forms a ‘flap’ over the active site with visible differences in the apex of this loop (residues 132–142; marked by a bracket in Figs. 4 ▸*b*–4 ▸*e*). In CjCASDC, this loop was not resolved, leaving a ten-residue gap in the model. In ClCASDC chain *A*, residues 138–139 were left unmodeled, although weak density is visible in the *mF*_o_ − *DF*_c_ map. In chain *B*, the β6/7 loop can be fully modelled, but the electron density is weaker, suggesting local mobility and a potential role in closing the active-site cavity to solvent during the catalytic cycle. The other regions of significant difference are within the β-barrel domain. AmCASDC has a 27-amino-acid insertion between α9 and β12. While this insertion does not participate in the dimeric interface, it does connect to α9, the specificity helix, and may play a role in tuning the position of this helix within the active site (marked with a circle in Fig. 4 ▸*d*). CjCASDC also has a smaller, six amino-acid-residue insertion between α9 and β12 which forms an additional β-strand connected to the specificity helix (marked with an oval in Fig. 4 ▸*b*).

*C. leptum* is a key constituent of the human gut microbiome. ClCASDC performs an essential metabolic task in the decarboxylation of carboxyspermidine to produce the polyamine spermidine. The structure of ClCASDC reported here is 0.5 Å higher in resolution than the previously described structure from *C. jejuni*. The active-site β6/7 loop is resolved, and a small shift is visible in Cys306, which may play a role in catalysis. CASDC is highly conserved among gut species although variation is present in two loops, one forming a ‘flap’ over the active site and the other connected to the specificity helix which is thought to select substrates based on length.

## Supplementary Material

PDB reference: *Clostridium leptum*carboxyspermidine decarboxylase, 9eaf

Supplementary Figures. DOI: 10.1107/S2053230X25000482/cha5002sup1.pdf

## Figures and Tables

**Table 1:** ClCASDC production

Source organism	*Clostridium leptum*
DNA source	Synthesized from sequence (NCBI EDO61992.1)
Expression vector	pET-28b (NdeI/XhoI)
Expression host	*Escherichia coli* BL21 (DE3)
Complete amino-acid sequence of the construct produced	MELPFSSLQTPCYVVDEALLERNLVILKQVIDRTGCKILLAQKAFSMFACYPLIGSYLNGTTASGLFEARLGKEEMGGETHIFSPAYREDEIDEILSLCDHVIFNSFSQWEKYKAKVLASGKSAGLRLNPEHSTQDHAIYDPCSPGSRLGITLKKFRPDLLDGIEGLHFHTLCEQDAAPLVETVAVVEEKFGPWLSQMKWLNFGGGHHITRPGYDIDALVSCVSRVQERYGVQVYLEPGEAVALNAGFLVSTVLDVLENSGNIAVLDTSAACHMPDVLEMPYRPPIAGGGGLGEKAYDYRLGGPTCLAGDVIGDYSFDEPLSPGSRVVFCDMAIYSMVKNNTFNGMNLPAIYLKKQDGSIQLVRKFGYEDFKTRLS

**Table 2:** Crystallization

Method	Sitting-drop vapour diffusion
Plate type	Intelli-Plate 24-4 (Art Robbins)
Temperature (K)	298
Protein concentration (mg ml^−1^)	4.5
Buffer composition of protein solution	25 m*M* HEPES pH 6.8, 150 m*M* NaCl, 10% glycerol
Composition of reservoir solution	0.04 *M* monopotassium phosphate, 16%(*w*/*v*) PEG 8000, 20%(*w*/*v*) glycerol
Volume and ratio of drop	1 µl:1 µl
Volume of reservoir (µl)	250

**Table 3:** Data collection and processing Values in parentheses are for the outer shell.

Diffraction source	Beamline 12-2, SSRL
Wavelength (Å)	0.9795
Temperature (K)	100
Detector	Dectris PILATUS 6M
Crystal-to-detector distance (mm)	213
Rotation range per image (°)	0.15
Total rotation range (°)	360
Exposure time per image (s)	0.2
Space group	*P*2_1_2_1_2_1_
*a*, *b*, *c* (Å)	63.57, 80.85, 140.54
α, β, γ (°)	90, 90, 90
Mosaicity (°)	0.06
Resolution range (Å)	38.85–1.41
Total No. of reflections	1758047
No. of unique reflections	133970
Completeness (%)	95.5 (70.7)
Multiplicity	13.1 (8.5)
〈*I*/σ(*I*)〉	12.9 (2.0)
*R*_r.i.m._ (%)	3.0 (33.5)
Overall *B* factor from Wilson plot (Å^2^)	10.63

**Table 4:** Structure solution and refinement Values in parentheses are for the outer shell.

Resolution range (Å)	38.85–1.41 (1.45–1.41)
Completeness (%)	95.5 (70.7)
σ Cutoff	*F* > 1.35σ(*F*)
No. of reflections, working set	131587 (6885)
No. of reflections, test set	1995 (104)
Final *R*_cryst_ (%)	15.0 (24.97)
Final *R*_free_ (%)	18.9 (30.42)
No. of non-H atoms	
Total	6447
Protein	5837
Ligand	31
Water	579
R.m.s. deviations	
Bond lengths (Å)	0.004
Angles (°)	0.730
Average *B* factors (Å^2^)	
Overall	14.4
Protein	13.3
Waters	24.6
PLP	11.7
Ramachandran plot	
Most favoured (%)	97.95
Allowed (%)	2.05

## References

[bb1] Adams, P. D., Afonine, P. V., Bunkóczi, G., Chen, V. B., Davis, I. W., Echols, N., Headd, J. J., Hung, L.-W., Kapral, G. J., Grosse-Kunstleve, R. W., McCoy, A. J., Moriarty, N. W., Oeffner, R., Read, R. J., Richardson, D. C., Richardson, J. S., Terwilliger, T. C. & Zwart, P. H. (2010). *Acta Cryst.* D**66**, 213–221. 10.1107/S0907444909052925 PMC2815670 20124702

[bb2] Almrud, J. J., Oliveira, M. A., Kern, A. D., Grishin, N. V., Phillips, M. A. & Hackert, M. L. (2000). *J. Mol. Biol.* **295**, 7–16. 10.1006/jmbi.1999.3331 10623504

[bb3] Chen, V. B., Arendall, W. B., Headd, J. J., Keedy, D. A., Immormino, R. M., Kapral, G. J., Murray, L. W., Richardson, J. S. & Richardson, D. C. (2010). *Acta Cryst.* D**66**, 12–21. 10.1107/S0907444909042073 PMC2803126 20057044

[bb4] Deng, X., Lee, J., Michael, A. J., Tomchick, D. R., Goldsmith, E. J. & Phillips, M. A. (2010). *J. Biol. Chem.* **285**, 25708–25719. 10.1074/jbc.M110.121137 PMC2919134 20534592

[bb5] Di Tommaso, P., Moretti, S., Xenarios, I., Orobitg, M., Montanyola, A., Chang, J. M., Taly, J. F. & Notredame, C. (2011). *Nucleic Acids Res.* **39**, W13–W17. 10.1093/nar/gkr245 PMC3125728 21558174

[bb6] Emsley, P., Lohkamp, B., Scott, W. G. & Cowtan, K. (2010). *Acta Cryst.* D**66**, 486–501. 10.1107/S0907444910007493 PMC2852313 20383002

[bb7] Holm, L. (2022). *Nucleic Acids Res.* **50**, W210–W215. 10.1093/nar/gkac387 PMC9252788 35610055

[bb8] Kabsch, W. (2010). *Acta Cryst.* D**66**, 125–132. 10.1107/S0907444909047337 PMC2815665 20124692

[bb9] Krissinel, E. & Henrick, K. (2004). *Acta Cryst.* D**60**, 2256–2268. 10.1107/S0907444904026460 15572779

[bb10] Krissinel, E. & Henrick, K. (2007). *J. Mol. Biol.* **372**, 774–797. 10.1016/j.jmb.2007.05.022 17681537

[bb11] Lee, D. F., Atencio, N., Bouchey, S., Shoemaker, M. R., Dodd, J. S., Satre, M., Miller, K. A. & McFarlane, J. S. (2023). *J. Biol. Chem.* **299**, 105033. 10.1016/j.jbc.2023.105033 PMC10413350 37437886

[bb12] Liu, C., Du, M.-X., Abuduaini, R., Yu, H.-Y., Li, D.-H., Wang, Y.-J., Zhou, N., Jiang, M.-Z., Niu, P.-X., Han, S.-S., Chen, H.-H., Shi, W.-Y., Wu, L., Xin, Y.-H., Ma, J., Zhou, Y., Jiang, C.-Y., Liu, H.-W. & Liu, S.-J. (2021). *Microbiome*, **9**, 119. 10.1186/s40168-021-01064-3 PMC8140505 34020714

[bb13] McCoy, A. J., Grosse-Kunstleve, R. W., Adams, P. D., Winn, M. D., Storoni, L. C. & Read, R. J. (2007). *J. Appl. Cryst.* **40**, 658–674. 10.1107/S0021889807021206 PMC2483472 19461840

[bb14] McPhillips, T. M., McPhillips, S. E., Chiu, H.-J., Cohen, A. E., Deacon, A. M., Ellis, P. J., Garman, E., Gonzalez, A., Sauter, N. K., Phizackerley, R. P., Soltis, S. M. & Kuhn, P. (2002). *J. Synchrotron Rad.* **9**, 401–406. 10.1107/s0909049502015170 12409628

[bb15] Mehta, P. K. & Christen, P. (2000). *Adv. Enzymol. Relat. Areas Mol. Biol.* **74**, 129–184. 10.1002/9780470123201.ch4 10800595

[bb16] Michael, A. J. (2016). *J. Biol. Chem.* **291**, 14896–14903. 10.1074/jbc.R116.734780 PMC4946907 27268252

[bb17] Pegg, A. E. & Michael, A. J. (2010). *Cell. Mol. Life Sci.* **67**, 113–121. 10.1007/s00018-009-0165-5 PMC2822986 19859664

[bb18] Ramos-Molina, B., Queipo-Ortuño, M. I., Lambertos, A., Tinahones, F. J. & Peñafiel, R. (2019). *Front. Nutr.* **6**, 24. 10.3389/fnut.2019.00024 PMC6426781 30923709

[bb19] Ray, S. S., Bonanno, J. B., Rajashankar, K. R., Pinho, M. G., He, G., De Lencastre, H., Tomasz, A. & Burley, S. K. (2002). *Structure*, **10**, 1499–1508. 10.1016/s0969-2126(02)00880-8 12429091

[bb20] Shah, P. & Swiatlo, E. (2008). *Mol. Microbiol.* **68**, 4–16. 10.1111/j.1365-2958.2008.06126.x 18405343

[bb21] Sugiyama, Y., Nara, M., Sakanaka, M., Gotoh, A., Kitakata, A., Okuda, S. & Kurihara, S. (2017). *Int. J. Biochem. Cell Biol.* **93**, 52–61. 10.1016/j.biocel.2017.10.015 29102547

