# Supplementary material for: Structure of Clostridium leptum carboxyspermidine decarboxylase and comparison to homologs prevalent within the human gut microbiome
Source: Acta Crystallogr F Struct Biol Commun. 2025 Jan 31;81(Pt 3):66–72. doi: 10.1107/S2053230X25000482 (PMC11866407; doi:10.1107/S2053230X25000482)
Supplement: Supplementary file 1 [file f-81-00066-sup1.pdf]

STRUCTURAL BIOLOGY
COMMUNICATIONS

Volume 81 (2025)

Supporting information for article:

Structure of *Clostridium leptum* carboxyspermidine decarboxylase and comparison to homologs prevalent within the human gut microbiome

Savannah J. Jones, Dawson J. Bell and Jeffrey S. McFarlane

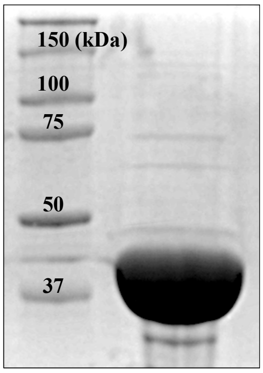

Figure S1 PAGE analysis of ClCASDC purification. ClCASDC has a M.W. of 41.372 kDa.

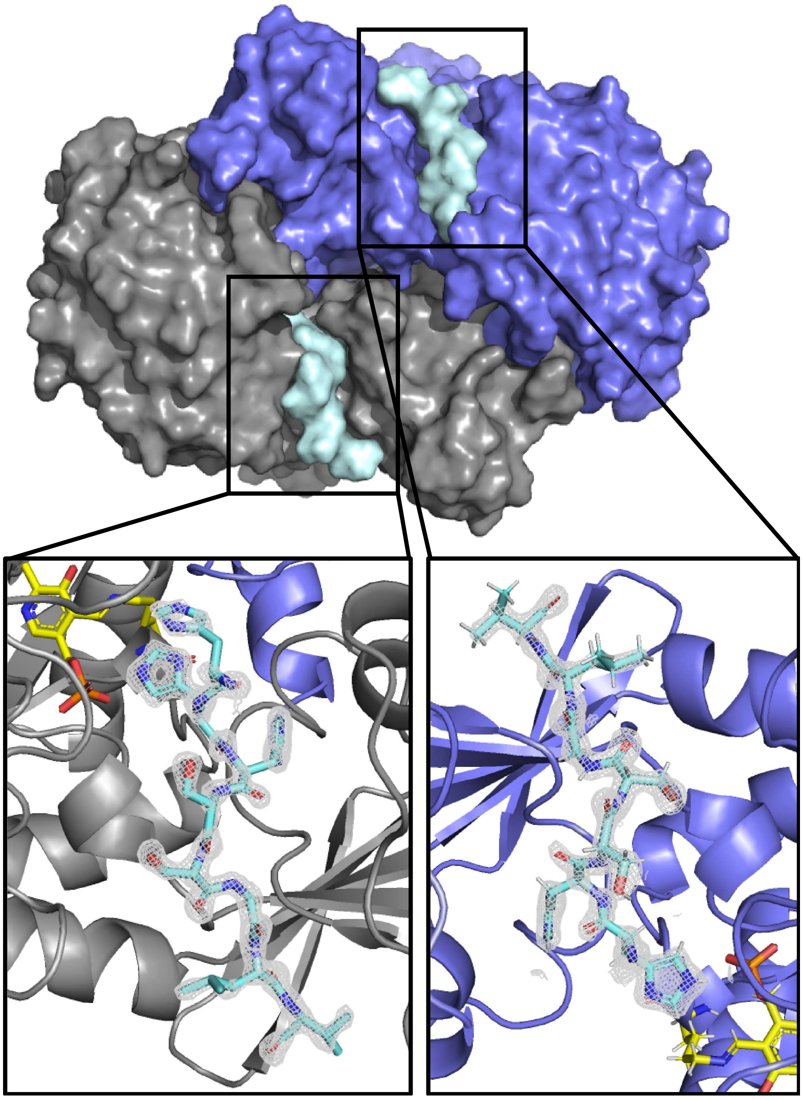

Figure S2 Position and electron density for hexahistidine affinity tags. Electron density is displayed as a 2mF_o - DF_c map contoured at 1.5 σ. The first two histidines (blue chain) or three histidines (grey chain) of the affinity tag are stabilized within the active site channel. Density for the remainder of the tag and linker was incomplete and left unmodeled.

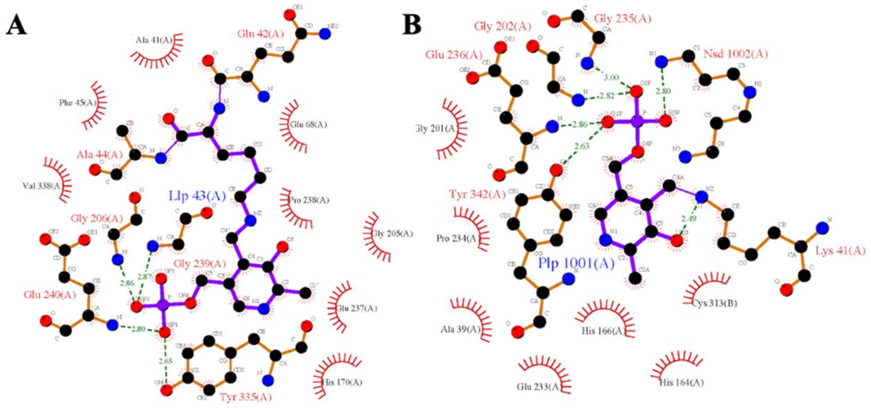

Figure S3 LigPlot active site comparison. **A.** Chain A active site of ClCASDC. **B.** Chain A active site of CjCASDC (PDB: 3N29).

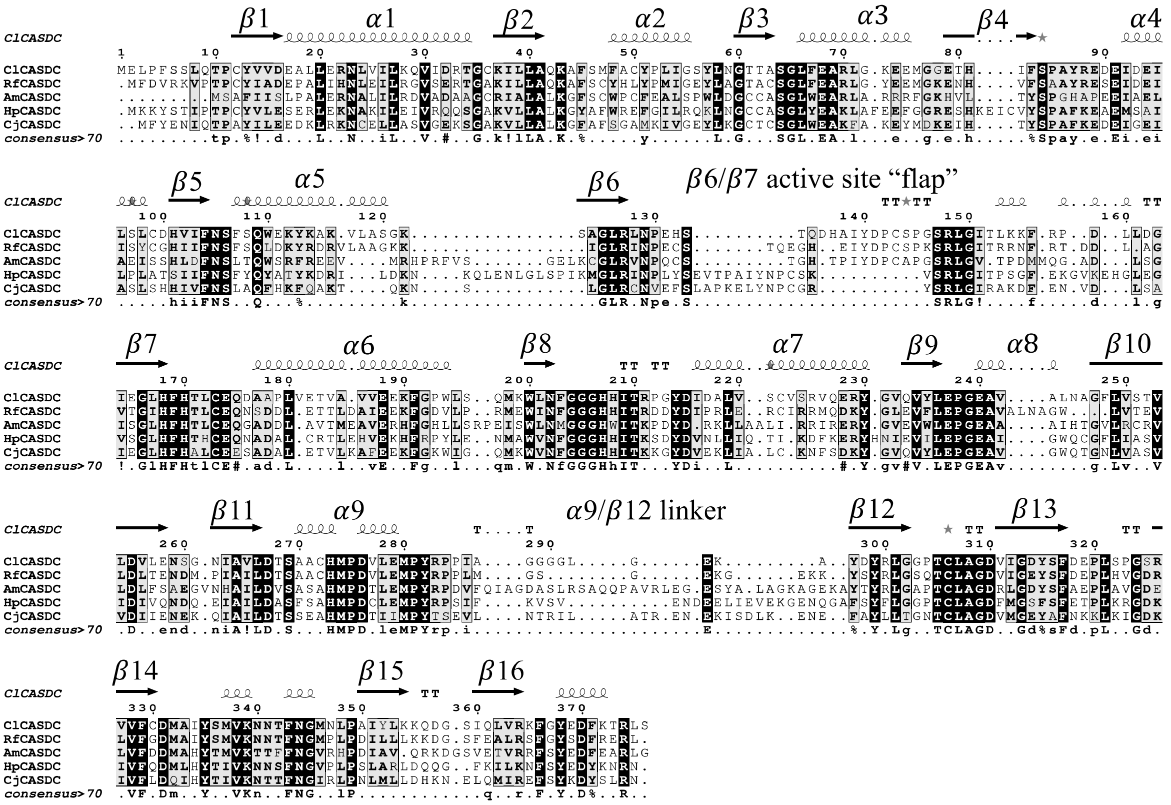

Figure S4 CASDC homolog alignment. CASDC homologs from *Clostridium leptu*m (ClCASDC), *Ruminococcus flavefaciens* (RfCASDC), *Akkermansia muciniphila* (AmCASDC), *Helicobacter pylori* (HpCASDC) and *Campylobacter jejuni* (CjCASDC) were aligned using the Espresso algorithm in T-Coffee with ClCASDC as the reference structure. Secondary structure alignment generated in ESPript 3.0.
